# Predicting lake dissolved organic carbon at a global scale

**DOI:** 10.1038/s41598-020-65010-3

**Published:** 2020-05-21

**Authors:** Kaire Toming, Jonne Kotta, Evelyn Uuemaa, Sebastian Sobek, Tiit Kutser, Lars J. Tranvik

**Affiliations:** 1 Limnology/Department of Ecology and Genetics, Uppsala University, Uppsala, Sweden; 2 Estonian Marine Institute, University of Tartu, Tallinn, Estonia; 3 Centre for Limnology, Estonian University of Life Sciences, Tartu, Estonia; 4 Institute of Ecology and Earth Sciences, University of Tartu, Tartu, Estonia

**Keywords:** Carbon cycle, Limnology

## Abstract

The pool of dissolved organic carbon (DOC), is one of the main regulators of the ecology and biogeochemistry of inland water ecosystems, and an important loss term in the carbon budgets of land ecosystems. We used a novel machine learning technique and global databases to test if and how different environmental factors contribute to the variability of *in situ* DOC concentrations in lakes. In order to estimate DOC in lakes globally we predicted DOC in each lake with a surface area larger than 0.1 km^2^. Catchment properties and meteorological and hydrological features explained most of the variability of the lake DOC concentration, whereas lake morphometry played only a marginal role. The predicted average of the global DOC concentration in lake water was 3.88 mg L^−1^. The global predicted pool of DOC in lake water was 729 Tg from which 421 Tg was the share of the Caspian Sea. The results provide global-scale evidence for ecological, climate and carbon cycle models of lake ecosystems and related future prognoses.

## Introduction

The necessity to understand and predict climate change and its impacts requires a solid comprehension of the global carbon cycle. Recent studies have demonstrated that lakes act as carbon hot spots in the landscape and thereby play a crucial role in global biogeochemical cycles, and significantly contribute to climate regulation^1,2^. Lakes process most of the carbon flux of terrestrial origin by degassing carbon dioxide (CO_2_) and methane (CH_4_) to the atmosphere^3–5^. Simultaneously, lakes and reservoirs are burying as much carbon in their sediments as is buried by the entire ocean over the same time period^6,7^. Hence, the intensity of carbon cycling in lakes appears to be disproportionately important relative to their small areal extent. Dissolved organic carbon (DOC) plays a significant role in lake ecosystems, and strongly regulates the carbon and energy cycle of inland waters^8^. Thus, it is important to identify which factors control the DOC concentration in lakes. Such factors might be (1) the physical properties of the catchment^9–13^; (2) morphometric characteristics of the lake^9^; (3) climatic properties of the particular region^9^; (4) autochthonous production of DOC inside the lake (5) abiotic and biotic mineralization of DOC^14,15^. All above mentioned characteristics might influence either separately or interactively the DOC concentration in lakes and can greatly affect global carbon cycle.

Although relationships between DOC and its main controlling factors in lakes have been investigated in many regional studies during the last decades, there is a limited knowledge at a global scale. Regional DOC models seem to have a limited potential in predicting DOC in other geographical areas of the world but the proportion of wetlands in the watershed and lake elevation are good predictors of lake DOC concentration across regions^16^. This is due to a hierarchical regulation of DOC in lakes, where climatic and topographic characteristics set the regional range of DOC concentrations, and catchment and lake properties then define the DOC concentration in each individual lake^9,17^. Among catchment, soil, and climate parameters, altitude, mean annual runoff, and precipitation have been negatively correlated with lake DOC, while conductivity, soil carbon density, and soil C: N ratio were positively related with lake DOC^9^. In another study the lake total organic carbon (TOC) was only weakly related to morphological characteristics whereas climatic controls described nearly half of variability in TOC^18^. Based on these relationships the estimated global mean concentrations and storage of TOC in lake water are 5.58 mg L^−1^ and 984 Tg^18^. Nevertheless, in these studies a large part of variability was left undescribed and therefore the model uncertainties were high. This is partly because linear regression techniques were used to define links between the DOC and its controlling factors whereas the relationships between the DOC and its controlling variables are often nonlinear^19^. Moreover, these relationships potentially involve many interactions that cannot be evaluated with traditional statistical relationships.

Here we used the novel machine learning technique Boosted Regression Trees (BRT)^20^, HydroLAKES v. 1.0^21,22^ and WorldClim v. 2.0^23^ databases, and a compilation of globally distributed lake DOC concentration data^9^ (1) to test if and how different factors contribute to the variability of *in situ* DOC concentrations in lakes (including reservoirs) and (2) based on the established relationships to predict DOC in lakes globally. Machine learning provides a theoretical framework that moves beyond traditional paradigm boundaries by learning from new data (rather than assuming an appropriate data model) and resolving simultaneously a broad range of functions (rather than oversimplifying situations). Therefore, we expect that the BRT modelling captures the complex patterns of DOC in lakes and particularly improves our understanding of the causes of that variation.

## Results

### The contribution of different factors to the variability of *in situ* DOC

Altogether 14 different variables were included in our study to predict DOC concentration of global lakes. After testing for multicollinearity, we excluded the variables “lake area” and “discharge”. When interpreting the results, however, we kept in mind that both shoreline length and total lake volume correlated with lake area as well as watershed area correlated with discharge.

**Figure 1:**
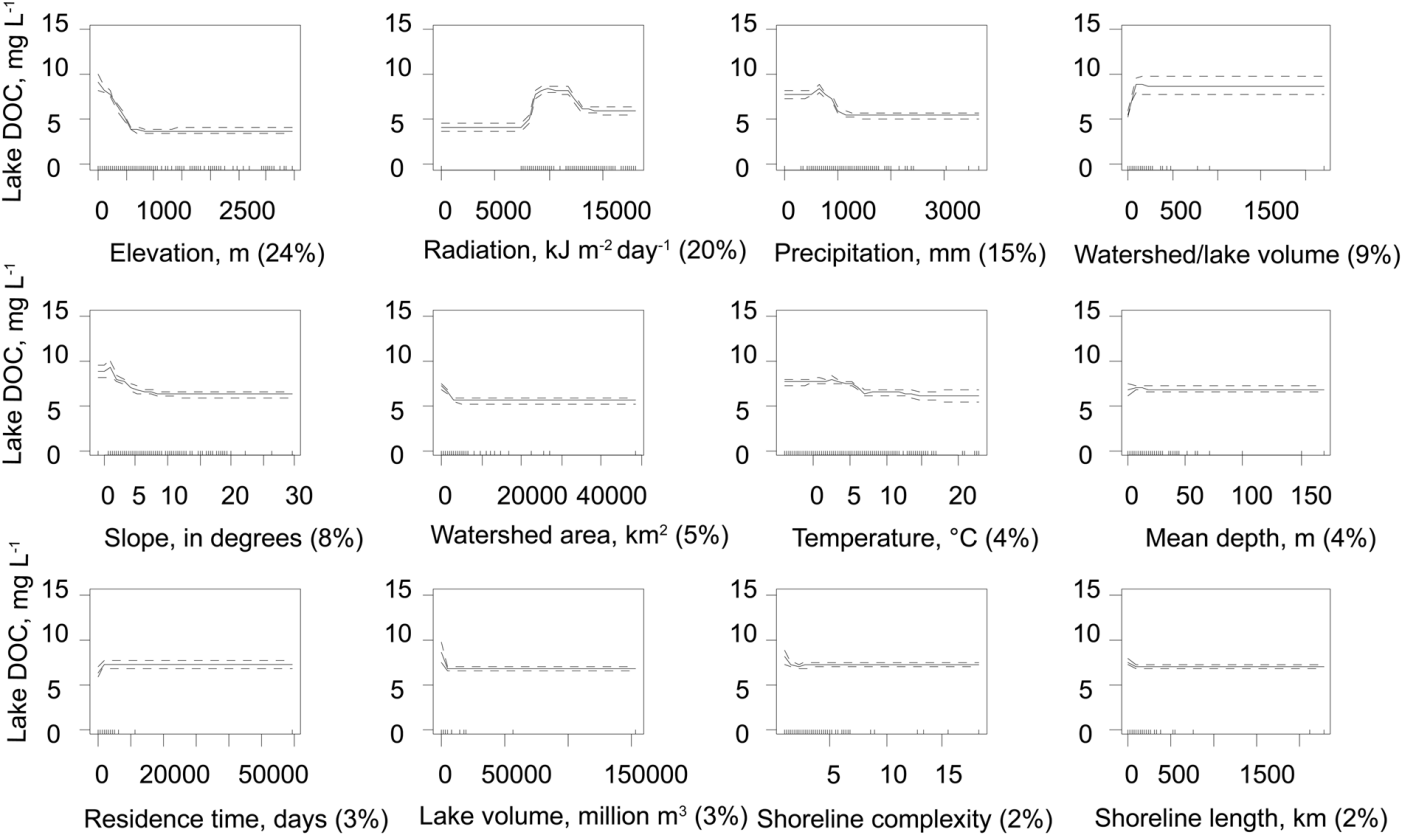
Partial dependence plots for Boosted Regression Trees analyses relating lake DOC concentrations to the different hydrological, meteorological and morphological variables. The figure shows the effect of an individual variable on the response when all other predictors are held at their mean values: positive fitted function values suggest that DOC respond positively and negative values suggest the opposite. The relative importance of each variable is shown in parentheses on the x-axis. Solid lines depict shapes of the response between environmental variables and DOC (the fitted function without a smoother) and dashed lines represent standard error values of partial dependence curves. Each tick mark at the bottom of each graph represents the 10th percentile of the data.

The BRT model described 78% of the variability in the lake DOC concentration. Among the studied environmental variables catchment physical properties (e.g. elevation of lake surface, watershed to lake volume ratio) and meteorological and hydrological features (surface solar radiation, precipitation) mostly explained to the variability of the lake DOC concentration (relative importance of those variables were 86%, Fig. 1) whereas lake morphometry played only a marginal role (relative importance of morphometrical variables were 14%, Fig. 1).

As expected, the observed relationships were not linear (Fig. 1) and showed different types of responses. Precipitation, elevation, discharge, slope, shoreline complexity and length as well as lake volume and area had negative effects on the DOC concentration. On the other hand, higher watershed area to lake volume ratio leads to higher DOC concentration. Solar radiation, lake depth, residence time and watershed area showed a unimodal response to the DOC concentration. Temperature was positively related to DOC concentration up to ~7 °C, but did not have any effect >7 °C.

### Predicting DOC concentration in lakes globally

**Figure 2:**
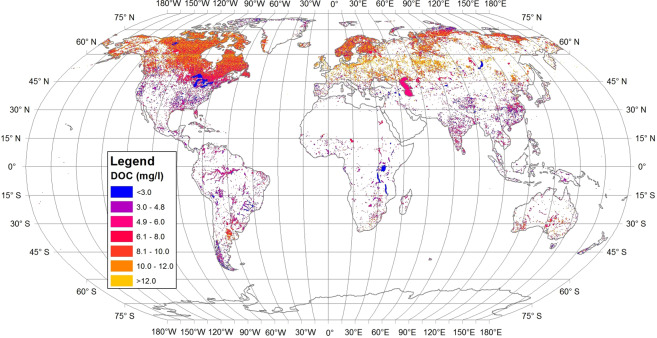
The DOC concentrations of global lakes.

The predictive model described in previous paragraph was used to predict DOC in lakes globally (Fig. 2). Volume weighted average DOC concentrations in global lake water were estimated to be 3.88 mg L^−1^ ranging from 0.0002 to 27.0 mg L^−1^ (Table 1). Area and volume of the most of lakes are smaller than 1 km^2^ and 1 km^3^, while the most of DOC can be found from lakes larger than 1 km^2^ and 1 km^3^. Consequently, larger lakes should count more than smaller lakes when calculating average DOC concentration of lakes globally. Therefore, we used a volume weighted average instead of arithmetic average when estimating the average DOC concentration in lakes.

**Table 1:** Predicted volume weighted average DOC concentrations (mg L^−1^) and total DOC pool (Tg) of global lakes by continent and for the whole world.

Continent	N	Mean DOC (mg L^−1^)	Min	Max	DOC pool (Tg)
Africa	15,958	2.13	0.0073	17.1	67.3
Asia	68,141	3.58	0.0002	20.4	31.0
Caspian Sea	1	5.57	—	—	421
Europe	281,874	2.29	0.0103	27.0	68.0
North America	993,837	3.31	0.0007	25.2	127
Oceania	13,479	3.46	0.0073	18.6	1.80
South America	54,024	3.55	0.0023	20.8	14.0
ALL	1,427,314	3.88	0.0002	27.0	729

The global storage of DOC in lake water was estimated to be 729 Tg. The majority of DOC was found in the Caspian Sea (421 Tg) while the European DOC pool was estimated to be 68 Tg – nearly the same than in Africa (67.3 Tg). The total predicted DOC pool in North America was 127 Tg.

The average DOC concentrations were higher in lakes with area and volume less than 100 km^2^ and 0.1 km^3^ respectively. Nevertheless, most of the global lakes DOC pool can be found in lakes larger than 1,000 km^2^ in area and 1,000 km^3^ in volume (Supplementary Table 1). Lakes with a mean depth less than 10 meters had average DOC concentrations higher than deeper lakes. On the other hand, total DOC amounts were higher in the deeper lakes (Supplementary Table 1). Higher shoreline complexity and longer shoreline length were associated with lower DOC concentrations. Lakes with shorter residence time had mostly higher mean DOC concentrations and lower DOC pool than the lakes with longer residence time.

The average DOC concentrations were somewhat higher in the lakes from the regions were annual mean air temperature is below 10 °C, also when we excluded the Caspian Sea (Supplementary Table 2). In the regions where annual mean precipitation remains below 500 mm, average DOC concentrations tended to be higher and these lakes constituted also the major part of the lake water DOC pool. Total DOC pools and the average DOC concentrations were higher in the regions where solar radiation is between 8,000 kJ m^−2^ day^−1^ and 15,000 kJ m^−2^ day^−1^ (Supplementary Table 2). When average long-term discharge flowing through the lake remained below 10 m^3^ s^−1^, the average DOC concentrations were higher than in lakes with higher discharges. Though, the total DOC pools were highest in the lakes with average long-term discharge over 1,000 m^3^ s^−1^ (Supplementary Table 2).

The highland lakes (elevation more than 100 m) had lower DOC concentrations and total DOC pools than lowland lakes (Supplementary Table 3). Average slope less than 10 degrees within a 100-meter buffer around the lake polygon favours higher lake DOC concentrations and total DOC pools. Lakes with watershed area less than 1,000 km^2^ had higher average DOC concentrations than lakes that were surrounded with larger watershed areas. It is probably related to the size of the lakes – smaller lakes have usually smaller watershed, but higher DOC concentrations than larger lakes. Additionally, the watershed area: lake volume ratio was positively related to the concentration of DOC and total DOC pools.

## Discussion

Machine learning lets us derive accurate relationships between environmental variables and DOC and thereby predict the contribution of different factors to the variability of *in situ* DOC. We predicted DOC for every lake on Earth which has a surface area of at least 0.1 km^2^ (ca 1.4 million lakes) taking into account^14^ different variables of lake morphometry, meteorology, hydrology and catchment without assumptions on model type.

Catchment physical properties (elevation of lake surface, watershed to lake volume ratio) and meteorological and hydrological features (surface solar radiation, precipitation) mostly explained the variability of the lake DOC concentration while lake morphometry (e.g. lake area and volume) was negligible in the global context. Hydrological and meteorological variables have been acknowledged as important controlling factors of DOC in surface waters in regional-scale studies^19,24,25^. In our study, the relative importance of meteorological and hydrological features to the overall variability in lake DOC concentration was 39%. Lower air temperature, solar radiation, precipitation and discharge values favour higher DOC concentrations in lakes (Supplementary Table 2). Temperature is positively related to DOC concentration up to ~7 °C, but did not have any effect >7 °C is similar to what Laudon *et al*.^24^ observed for stream DOC across regions. They suggest, that at higher temperatures the soil DOC becomes production limited meaning that together with large litter input and organic matter production high mineralization rate dominates^26^. Additionally, higher air temperature and solar radiation enhance the biological and photochemical activity and thereby rise the rate of DOC decomposition and bioavailability leading to reduction of DOC concentrations in surface waters^27,28^. DOC concentrations in lakes could be lower also in the regions of high precipitation and discharge due to low DOC pools in catchment soils and due to the dilution effect^29,30^. In regions with very low precipitation and discharge, evaporation can lead to very high DOC concentration^31^. In addition to the climatic variables, catchment characteristics appeared to be also very important predictors of lake water DOC concentration (relative importance of all catchment features was 46%). The most important predictor of DOC among catchment characteristics and also among all variables was elevation. Highland lakes have lower DOC concentrations and total DOC values than the lowland lakes (Supplementary Table 3). Sobek *et al*.^9^ showed that the catchments at higher altitudes experience more precipitation, which leads to higher area-specific runoff associated with low soil carbon density and low lake DOC concentrations. Similarly, alpine lakes and high plateaus experience high values of solar radiation that will decrease the in-lake DOC concentration through active photochemical decomposition^32^. Additionally, the catchments slopes tend to be hillier at higher altitudes^9^ and as seen in Supplementary Table 3, steeper slopes result in lower DOC concentrations in lakes, possibly related to organic soil horizons being thinner on steeper slopes^33^ and to a smaller proportion of wet soils compare to flatter catchments^34^. Similarly, Musolff *et al*.^35^ found that the long-term median DOC concentration in the catchment is well predicted by the 90th percentile of the distribution of the topographic wetness index (0.9 P TWI) over the entire catchment area and catchments with a high 0.9 P TWI and low slopes generally exhibited high DOC concentrations. Thus, the close relationships of the altitude with climatic and soil properties makes it a good predictor for lake water DOC concentration at a global scale.

It is well known that nutrients determine the trophic state of lakes. Therefore, it would be desirable to incorporate them into our model in order to improve the predictions. However, nutrient data is currently not available at the global scale and therefore cannot be incorporated to our modelling framework. On the other hand, the used variables (e.g. temperature, radiation, precipitation, discharge, mean depth, etc.) are highly linked to the trophic state of lakes and thereby its signal is indirectly captured in our model. Though, we acknowledge that without global data of nutrients trophic state is taken into account to a limited extent.

Globally, reservoirs (both large and small) were estimated to comprise roughly 9% of the total lake and reservoir surface area^5,7^. Many reservoirs possess properties that are associated with low DOC concentration (e.g. high shoreline complexity, large volume), but also often have large catchment areas, resulting in relatively short water residence time and implying high DOC concentrations^36,37^. While we cannot exclude that the presence and abundance of reservoirs may affect the patterns observed in our study, we argue that their relatively low contribution to total area, as well as the potentially counteracting effects on DOC concentrations, are unlikely to lead to a systematic effect.

The significant contribution of catchment physical properties, lake morphometric characteristics, and meteorological and hydrological features to the variability of lake DOC allowed us to predict reliably lake DOC values globally from these drivers. Since most of the global lakes are located in Europe and North America, expectedly also the total DOC pool in lake water appeared to be highest in those continents. However, the DOC pool of Africa is very similar to Europe due to the very high volume of water in African rift valley lakes. Higher mean DOC concentrations in Europe and North America compared to other continents can be explained by the meteorological and hydrological features and the lake morphometric characteristics of these regions that support higher DOC concentrations. Clear differences in DOC concentrations appeared between large and small lakes, between high volume and low volume lakes, and between deep and shallow lakes. Nevertheless, in accordance with previous studies^9,16,38^ none of these parameters were among the main predictors of DOC in lakes globally. Similarly, the residence time of lake water, which is associated with in-lake rates of photochemical and biological degradation of DOC^39^, was not among the main predictors of the concentrations of lake water DOC globally. This supports the view that lake properties are important predictors of the DOC concentration regionally^9^ whereas climate and catchment properties are more important globally.

A significant fraction of the global lake DOC pool is in the Caspian Sea. Our prediction for the mean DOC was 5.57 mg L^−1^. There is a recent publication^40^ indicating that the DOC may vary between 5.93 mg L^−1^ and as high as 19.2 mg L^−1^ in some coastal regions of the Caspian Sea. This suggests that our Caspian Sea DOC pool estimate may be rather conservative. There is very little *in situ* data available from the Caspian Sea and there is a strong need to get more information in order to improve global lake DOC pool estimates.

Our estimates on the DOC concentrations and the global pool of DOC in lake water are somewhat lower than previous results of total organic carbon (TOC) obtained by Chen *et al*.^18^. They used in their TOC estimates a global data set that covers approximately 8,300 lakes from 68 countries/regions spanning six continents and found that the global mean TOC concentration is 5.578 mg L^−1^ and the pool in lake water is 984 Tg. After converting Chen’s *et al*.^18^ TOC values to DOC by multiplying 0.9^41^, their estimate of the global lake DOC pool is 17.7% bigger than obtained in current study, and the difference is even bigger when comparing the mean DOC concentrations (22.7%). Even if we count lakes that are smaller than 0.1 km^3^ it will only slightly affect (less than 1%) the total DOC pool and the estimates of the mean DOC concentrations due to the low total volume of smaller lakes^42^. We argue that our study more accurately predicts lake DOC concentration globally since it incorporates, for each lake, the complex relationships between multiple meteorological, hydrological, topographical, morphometrical variables and the DOC concentration. Moreover, our model benefited from the inclusion of important variables such as elevation, the ratio of the watershed area to lake volume, water residence time and global meteorological data with a very good spatial resolution (1 km). As in other predictive modelling, BRT is a process that uses data mining and the laws of probability to predict values of dependent variable. Thus, one of the biggest challenges of predictive modelling is acquiring the right training data when developing algorithms, because data quality defines the accuracy of prediction. Although we used the best free data sources available, some regions were better sampled than others (see environmental data distribution in Fig. 1) and in these regions the established functions are more detailed and precise. Therefore, dedicated sampling efforts in the under-sampled regions of the world are necessary to improve predictive models.

Our results show that the global lake DOC pool (0.729 Pg) is a small fraction of the DOC pool in the global surface ocean (700 Pg), as well as carbon pool in vegetation (450–650 Pg) and soils (1500–2400 Pg)^43^. However, lakes are extremely active sites for transport, transformation, and storage of considerable amounts of carbon received from the terrestrial environment and therefore have an effect on the global carbon cycle that is disproportional to their spatial extent^44^. For example, lakes and other inland waters bury 0.2 Pg of carbon in their sediments and outgas 1.0 Pg CO_2_ and CH_4_ to the atmosphere at global scales annually^2,43^. Inland waters are thus burying as much carbon in their sediments as is buried by the entire ocean over the same time period.

Thus, lakes may act both as sinks and sources for carbon. In many lakes, especially within the cool boreal climate region, heterotrophic processes prevail and lakes act as sources of CO_2_^45^. The reason for that is the large amount of organic carbon runoff from the catchment and discharge to the lakes that increases the respiration rate of the lake ecosystem to a level that exceeds photosynthetic carbon sequestering by phytoplankton and aquatic plants. Seekell *et al*.^46^ evaluated the relationship between DOC and whole-lake primary production in lakes and found a threshold DOC concentration (4.8 mg L^−1^), below which the DOC-primary production relationship is positive, and above which the relationship is negative. In our study, 89.5% of lakes were above and 10.5% of lakes were below threshold indicating that in the most of the global lakes DOC limits the primary production. However, this is still a growing area of research, and further research is needed to conclude whether DOC is suppressing gross primary production in the most of the lakes.

In conclusion, we used the best available global data, numerous *in situ* DOC measurements from various regions and a powerful machine learning algorithm to describe complex interactions between the lake DOC and its environmental predictors and to reliably predict the lake DOC pool globally. As such the results improve our understanding on the role of different environmental variables in the lake DOC and the global carbon cycle in general. Consequently, our predictions can be regarded as an important milestone providing a valuable input for global ecological, climate and carbon cycle models and related future prognoses.

The DOC concentrations and pools reported here are not static, but may change in response to changing environments. For example, boreal and temperate lakes of NW Europe and NE North America have had increasing concentrations of DOC over several decades, possibly due to recovery from acidification of soils with concomitant increased export of DOC to lakes^47^, but it may also be a result of afforestation or other land use change^48^ or changing runoff patterns^49^. There are also spatial variations in DOC concentrations within lakes. However, we were not able to consider in detail spatial and temporal variability of DOC in our global study due to data unavailability. Therefore, further development will be critical in this field in making more accurate predictions about lake DOC pool globally. For example, two Sentinel-2 satellites with 10 m spatial resolution and 2–5 days revisit time should enable mapping spatial variability of DOC concentration within lakes as well as seasonal variations^50^. However, global validation is needed for remote sensing algorithms as the existing DOC retrieval ones have been validated only at regional scale.

## Methods

### Data

Measured DOC concentrations (mg L^−1^) of lakes were obtained from Sobek *et al*.^9^. DOC concentrations from lakes larger than 0.1 km^2^ were used (1306 lakes in total). Additionally, DOC concentration data of lake water available in literature for large lakes Baikal^51^, Malawi^52^, Superior^53^, Michigan^54^, Ontario, Huron and Erie^55^ were used.

Global climate data, i.e. annual mean air temperature (°C), precipitation (mm), and solar radiation (kJ m^−2^ day^−1^) were obtained from WorldClim v. 2.0 23. Data with a spatial resolution of 30 s (~1 km^2^) were used.

Data of global lakes and reservoirs were obtained from HydroLAKES v. 1.0 21. The HydroLAKES database includes all lakes and reservoirs with a surface area of at least 0.1 km^2^. It comprises 1,427,688 individual lakes containing both freshwater and saline lakes, including the Caspian Sea, as well as human-made reservoirs and regulated lakes 21. Furthermore, HydroLAKES contains additional information about different water and catchment characteristics. The following parameters from HydroLAKES database were used in the current study: elevation of lake surface (m above sea level), average slope within a 100 meter buffer around the lake polygon (degrees), long-term discharge flowing through the lake, m^3^ s^−1^, average residence time of the lake water (days), average lake depth (m), shoreline complexity (measured as the ratio between shoreline length and the circumference of a circle with the same area), total lake or reservoir volume, (million m^3^), area of the watershed associated with the lake (km^2^), lake surface area (km^2^), length of shoreline (km). For more detailed information please see HydroLAKES Technical Documentation Version 1.0 22. Additionally, to HydroLAKES parameters, the ratio between the watershed area and lake volume was calculated. Continents are delimited based on HydroLAKES database as follows: Europe includes all of Russia, Asia includes Middle East and Turkey, North America includes Mexico, the Caribbean and Central America, Oceania includes Australia, New Zealand, Micronesia, Melanesia and Polynesia.

### Data analysis

Data of Sobek *et al*.^9^, HydroLAKES v.1.0 21 and WorldClim v. 2.0 23 were compiled using ArcGIS 10.4.1. Boosted Regression Trees (BRT; R 3.2.2. for Windows)^20^ was used to quantify relationships between environmental variables and the measured lake DOC values and then use the established relationships to predict the lake DOC values globally. In contrast to traditional regression techniques, BRT avoids starting with a data model, and rather uses an algorithm to learn the relationship between the response and its predictors^20^. BRT was first used to test if and how different factors (predictors) contribute to the variability of measured DOC in lakes (training data). Then, BRT was used to predict DOC in each individual lake (with a surface area of at least 0.1 km^2^) globally based on the predictive model created from the first step (model application). In fitting a BRT, the learning rate and the tree complexity must be specified. The optimum model was selected based on model performance, with learning rates, number of trees, and interaction depth set at 0.001, 3000, and 5, respectively. Model performance was evaluated using the cross-validation statistics calculated during model fitting^56^. For more details on the BRT modeling see Kotta *et al*.^57^. Standard errors for the predictions and pointwise standard errors for the partial dependence curves, produced by R package “pdp“^58^, were estimated using bootstrap (100 replications). Multicollinearity can be an issue with the BRT modelling when answering if and when environmental variables are of ecological interest. Thus, prior to modelling, the Pearson correlation analysis between all environmental variables was run in order to avoid situations of including highly correlated variables into the modelling. The correlation analysis showed that most variables were only weakly intercorrelated at r < 0.5. However, for some variables the values were far above the critical threshold when collinearity begins to severely distort model estimation and subsequent prediction^59^. Specifically, lake area correlated with both shore length (0.81) and total volume (0.9) as well as watershed area correlated with discharge (0.91). Thus, in the revised model we excluded the variables lake area and discharge.

## Supplementary information

Supplementary Tables.

## Data Availability

Global climate data were obtained from WorldClim v. 2.0. Data can be found here: http://worldclim.org/version2 Data of global lakes were obtained from HydroLAKES v. 1.0. Data can be found here: https://www.hydrosheds.org/pages/hydrolakes Other data can be found at 10.5281/zenodo.3452123.
