# Supplementary material for: Predicting lake dissolved organic carbon at a global scale
Source: Sci Rep. 2020 May 21;10:8471. doi: 10.1038/s41598-020-65010-3 (PMC7242472; doi:10.1038/s41598-020-65010-3)
Supplement: Supplementary file 1 — Supplementary Tables. [file 41598_2020_65010_MOESM1_ESM.pdf]

SUPPORTING INFORMATION FOR:

Predicting lake dissolved organic carbon at a global scale

Toming, Kaire*; Kotta, Jonne; Uuemaa, Evelyn, Sobek, Sebastian; Kutser, Tiit; Tranvik, Lars J.

* Correspondence and requests for materials should be addressed to K. Toming (email:

kaire.toming.001@ut.ee)

This PDF includes:

Supporting Tables 1 – 3

Table 1. Predicted volume weighted average DOC concentrations (mg L^{-1}) and total DOC pools (Tg) of global lakes by classes of morphometry (area, volume, depth, shoreline length and development) and residence time.

**Lake area (km^2)**	**N**	**DOC (mg L^{-1})**			**DOC pool (Tg)**
		**Mean**	**Min**	**Max**	
= 371,000 (Caspian Sea)	1	5.57	-	-	421
>10,000 and ≤ 100,000	17	1.96	1.0000	8.04	162
>1,000 and ≤ 10,000	160	4.58	2.1620	11.9	56.1
>100 and ≤ 1,000	1,526	4.15	0.0769	12.8	34.7
>10 and ≤ 100	14,963	4.88	0.0003	18.5	24.2
>1 and ≤ 10	166,590	7.03	0.0022	21.8	19.0
≥0.1 and ≤ 1	1,244,112	8.36	0.0002	27.0	11.0
**Lake volume (km^3)**	**N**	**DOC (mg L^{-1})**			**DOC pool (Tg)**
		**Mean**	**Min**	**Max**	
= 78,200 (Caspian Sea)	1	5.57	-	-	421
>10,000 and ≤ 25,000	3	1.70	1.2000	2.42	92.0
>1,000 and ≤ 10,000	9	2.30	1.0000	6.49	64.1
>100 and ≤ 1,000	33	4.54	0.7184	8.15	40.6
>10 and ≤ 100	296	4.60	0.0293	11.9	36.1
>1 and ≤ 10	2,087	4.45	0.0003	12.8	26.0
>0.1 and ≤ 1	11,377	5.54	0.2400	17.3	17.7
≤ 0.1	1,413,525	7.98	0.0002	27.0	31.0
**Lake mean depth (m)**	**N**	**DOC (mg L^{-1})**			**DOC pool (Tg)**
		**Mean**	**Min**	**Max**	
= 758 (Lake Baikal)	1	1.22	-	-	28.7
>100 and ≤ 600 (with Caspian Sea)	241	4.22	0.0293	9.43	513
>100 and ≤ 600 (without Caspian Sea)	240	2.00	0.0293	9.43	91.4
>10 and ≤ 100	35,219	3.80	0.0003	15.4	135
>1 and ≤ 10	136,0534	7.74	0.0002	27.0	42.0
≤ 1	31,318	6.81	0.7856	23.1	0.40
**Shoreline length (km)**	**N**	**DOC (mg L^{-1})**			**DOC pool (Tg)**
		**Mean**	**Min**	**Max**	
= 15,829 (Caspian Sea)	1	5.57	-	-	421
>1,000 and ≤ 10,000	176	2.15	1.0000	11.9	188
>100 and ≤ 1,000	3,900	4.24	0.0066	17.3	71.1
>10 and ≤ 100	100,098	5.45	0.0003	21.5	32.0
>2 and ≤ 10	922,122	8.15	0.0002	27.0	15.0
≤ 2	401,017	8.00	0.0037	25.6	2.00
**Shoreline complexity**	**N**	**DOC (mg L^{-1})**			**DOC pool (Tg)**
		**Mean**	**Min**	**Max**	
>25 and ≤ 55	24	6.45	3.81	9.41	5.86
>10 and ≤ 25	730	4.27	0.0293	14.4	39.5
>5 and ≤ 10 (with Caspian Sea)	7,970	4.84	0.0092	19.3	488
>5 and ≤ 10 (without Caspian Sea)	7,969	2.65	0.0092	19.3	66.9
>2 and ≤ 5	293,284	2.25	0.0003	23.0	158
≤ 2	112,5307	5.66	0.0002	27.0	37.0
**Residence time (days)**	**N**	**DOC (mg L^{-1})**			**DOC pool (Tg)**
		**Mean**	**Min**	**Max**	
>100,000 and ≤ 3,000,000 (with Caspian Sea)	557	4.14	0.2209	15.2	505
>100,000 and ≤ 3,000,000 (without Caspian Sea)	556	1.81	0.2209	15.2	83.4
>10,000 and ≤ 100,000	42,195	1.95	0.0567	19.0	67.7
>1,000 and ≤ 10,000	436,207	4.56	0.0254	20.8	84.0
>100 and ≤ 1,000	573,085	5.41	0.0003	26.0	47.0
>10 and ≤ 100	252,824	6.57	0.0022	27.0	12.0
≤ 10	122,443	5.41	0.0002	21.3	14.0

Table 2. Predicted volume weighted average DOC concentrations (mg L^{-1}) and total DOC pool (Tg) of global lakes by meteorological and hydrological characteristics (air temperature, precipitation, solar radiation, discharge).

**Air temperature (°C)**	**N**	**DOC (mg L^{-1})**			**DOC pool (Tg)**
		**Mean**	**Min**	**Max**	
>20 and ≤ 30	87,011	2.23	0.0002	18.0	75.1
>10 and ≤ 20 (with Caspian Sea)	63,748	5.46	0.0022	23.8	443
>10 and ≤ 20 (without Caspian Sea)	63,747	3.87	0.0022	23.8	21.2
>0 and ≤ 10	269,917	2.83	0.0007	27.0	100
>-10 and ≤ 0	638,462	2.54	0.0037	25.2	88.0
≤ -10	368,116	7.69	0.0103	24.7	23.0
**Precipitation (mm)**	**N**	**DOC (mg L^{-1})**			**DOC pool (Tg)**
		**Mean**	**Min**	**Max**	
>1,000 and ≤ 3,500	172,132	1.95	0.0022	18.7	31.9
>500 and ≤ 1,000	452,006	2.84	0.0002	27.0	153
>250 and ≤ 500 (with Caspian Sea)	491,047	4.61	0.0003	24.7	505
>250 and ≤ 500 (without Caspian Sea)	491,046	2.47	0.0003	24.7	84
>100 and ≤ 250	298,732	4.80	0.0037	24.3	36.0
≤ 100	10,066	5.17	0.0670	19.9	2.90
**Solar radiation (kJ m^{-2} day^{-1})**	**N**	**DOC (mg L^{-1})**			**DOC pool (Tg)**
		**Mean**	**Min**	**Max**	
>15,000 and ≤ 20,000	106,917	2.23	0.0003	18.6	87.1
>8,000 and ≤ 15,000 (with Caspian Sea)	1,218,200	4.31	0.0002	27.0	635
>8,000 and ≤ 15,000 (without Caspian Sea)	1,218,199	2.98	0.0002	27.0	213
≤ 8,000	84,002	3.98	0.0037	16.0	2.50
**Discharge (m^3 s^{-1})**	**N**	**DOC (mg L^{-1})**			**DOC pool (Tg)**
		**Mean**	**Min**	**Max**	
>1,000 and ≤ 15,000 (with Caspian Sea)	1,183	3.73	0.0002	14.5	584
>1,000 and ≤ 15,000 (without Caspian Sea)	1,182	2.01	0.0100	14.5	163
>100 and ≤ 1,000	6,384	4.16	0.0003	16.2	48.3
>10 and ≤ 100	24,997	3.62	0.0007	18.4	34.9
>1 and ≤ 10	91,012	5.63	0.0073	20.4	20.5
≤ 1	1,303,705	6.68	0.0037	27.0	41.0

Table 3. Predicted volume weighted average DOC concentrations (mg L^{-1}) and total DOC pools (Tg) of global lakes by catchment characteristics (elevation, slope, watershed area, watershed area/lake volume ratio).

Elevation (m)	N	DOC (mg L^{-1})			DOC pool (Tg)
		Mean	Min	Max	
>1,000 and ≤ 6,000	37,946	2.32	0.0066	12.5	20.8
>100 and ≤ 1,000	925,316	2.36	0.0002	24.0	216
>10 and ≤ 100	360,679	5.77	0.4641	27.0	41.0
>1 and ≤ 10	74,537	6.76	0.1058	23.4	6.90
≤ 1 (with Caspian Sea)	28,835	5.60	0.6890	23.3	444
≤ 1 (without Caspian Sea)	28,834	6.14	0.6890	23.3	22.7
**Slope (degrees)**	**N**	**DOC (mg L^{-1})**			**DOC pool (Tg)**
		**Mean**	**Min**	**Max**	
>30 and ≤ 60	591	3.07	0.1596	11.9	0.02
>10 and ≤ 30	47,771	2.76	0.0002	19.3	7.50
>1 and ≤ 10	1,038,226	5.52	0.0066	27.0	64.0
≤ 1 (with Caspian Sea)	340,726	3.79	0.7856	24.2	658
≤ 1 (without Caspian Sea)	340,725	2.41	0.7856	24.2	237
**Watershed area (km^2)**	**N**	**DOC (mg L^{-1})**			**DOC pool (Tg)**
		**Mean**	**Min**	**Max**	
>100,000 and ≤ 3,000,000 (with Caspian Sea)	1,368	3.71	0.0002	14.5	591
>100,000 and ≤ 3,000,000 (without Caspian Sea)	1,367	2.02	0.0002	14.5	169
>10,000 and ≤ 100,000	7,464	4.51	0.0003	17.1	59.3
>1,000 and ≤ 10,000	28,238	3.92	0.0007	18.0	32.4
>100 and ≤ 1,000	100,909	5.64	0.0140	21.1	20.4
≤ 100	1,289,330	3.07	0.0037	27.0	26.0
**Watershed area/ lake volume**	**N**	**DOC (mg L^{-1})**			**DOC pool (Tg)**
		**Mean**	**Min**	**Max**	
> 100	114,101	6.68	0.0002	24.0	2.00
>10 and ≤ 100	281,016	6.66	0.0022	27.0	18.0
>1 and ≤ 10	666,582	5.79	0.0003	25.1	54.0
≤ 1 (with Caspian)	365,610	3.73	0.0037	16.5	655
≤ 1 (without Caspian)	365,609	2.34	0.0037	16.5	233
